# Genome-wide identification and expression analysis of the AS2/LOB gene family in physic nut

**DOI:** 10.3389/fpls.2024.1504093

**Published:** 2024-12-03

**Authors:** Yuehui Tang, Xiaohui Wang, Jiayu Feng, Yaoyao Wang, Tengfei Liu, Xinxin Bao

**Affiliations:** College of Life Science and Agronomy, Zhoukou Normal University, Zhoukou, Henan, China

**Keywords:** AS2/LOB gene family, abiotic stress, physic nut, expression pattern, phylogenetic tree

## Abstract

**Introduction:**

AS2/LOB genes, a class of transcription factors ubiquitously existing in plants, are vital for plant growth, development, and stress tolerance. Despite the availability of the physic nut genome, information regarding the expression profiles and evolutionary histories of its AS2/LOB genes remains scarce.

**Methods:**

An elaborate exploration of the AS2/LOB gene family was conducted, including phylogeny, exon-intron structure, chromosomal location, conserved domain characteristics, conserved motifs, promoter cis-acting elements, protein interaction, and expression profiles under normal growth and abiotic stress conditions.

**Results:**

In this study, 28 AS2/LOB genes (JcASLs) were identified in the physic nut genome. Phylogenetic analysis, based on homologs from Arabidopsis, classified the 28 *JcASLs* genes into two groups (calss I and II). Chromosome localization indicated that the 28 *JcASLs* genes were unevenly distributed across nine chromosomes. RNA-seq and qRT-PCR results revealed that the majority of the 28 *JcASLs* genes exhibited differential expression in tissues such as roots, cortex stems, leaves, and seeds. Notably, *JcASL8* and *JcASL13* were exclusively expressed in seeds, and 16 *JcASLs* genes responded to drought and salt stress at least at one time point under at least one treatment condition.

**Discussion:**

These results establish a basis for future investigations into the molecular mechanism by which the *JcASLs* genes regulate physic nut's response to drought and salt stress and their role in modulating the growth and development of physic nut.

## Introduction

Abiotic stresses, such as drought and salt stress, are major limiting factors that exert significant influence on plant growth and development. Plants have the ability to recognize these abiotic stresses and execute a series of signal transductions through interactions with defense-related proteins, thereby activating or inhibiting the transcription of downstream target genes. Among them, transcription factors like HD-Zip, WRKY, MYB, and AS2/LOB play a crucial role in regulating plant responses to abiotic stress (Li et al., 2022; Ma and Hu, 2024; Rong et al., 2024; Zhang et al., 2024).

AS2/LOB transcription factors constitute a family of plant-specific transcription factors, containing a highly conserved AS2/LOB domain composed of a C-motif (CX2CX6CX3C) with four cysteine residues, a conserved glycine residue, and a leucine zipper-like sequence (Iwakawa et al., 2002; Matsumura et al., 2009). The C motif is primarily indispensable for DNA binding activity, the glycine residue is indispensable for the function of AS2/LOB, and the leucine zipper-like sequence is mainly responsible for protein dimerization (Xu et al., 2016). Based on the deduced amino acid sequence of the AS2/LOB conserved domain, the AS2/LOB family is classified into two groups: Class I and Class II, and Class I further divided into Class Ia and Class Ib (Iwakawa et al., 2002). Class I proteins contain both the conserved C- motif and the leucine-zipper-like sequence. In contrast, class II proteins contain only the conserved C-motif. Additionally, a variable C-terminal region emerges immediately following the conserved leucine zipper-like sequence of the LOB domain of AS2/LOB proteins (Kim and Kim, 2012). This variable C-terminal region is capable of regulating the expression of downstream genes (Kim and Kim, 2012).

Since the initial discovery of the first AS2/LOB family gene, *AS2*, from Arabidopsis (Iwakawa et al., 2002), a growing number of AS2/LOB family members have been identified, and their biological functions have been gradually elucidated in various plants. For instance, the *LaLBD37* gene regulates the bulblet development in lily (*Lilium brownii*) (Hou et al., 2024), while *AtASL4*/*AtLOB* and *AtASL5*/*AtLBD12* are crucial for the development of meristem lateral organs in Arabidopsis (Chalfun-Junior et al., 2005; Nakazawa et al., 2003). ASYMMETRIC LEAVES2 (AS2) serves as a key regulator of leaf adaxial-abaxial partitioning by directly suppressing the expression of the Arabidopsis leaf abaxial determination gene *ETTIN/AUXIN RESPONSE FACTOR3* (*ETT/ARF3*) (Iwakawa and Takahashi, 2020). The CmLBD2 protein positively regulates pollen development of *Chrysanthemum morifolium* by activating the expression of downstream target gene *CmACOS5* (Teng et al., 2021). *ASL39*/*LBD37* has been identified as a repressor of anthocyanin metabolism regulation in Arabidopsis (Rubin et al., 2009), and *ASL38*/*LBD41* plays a specific role in adaxial cell fate in Arabidopsis lateral organs (Wang et al., 2015). Moreover, overexpression of *CsLBD37* leads to transgenic Arabidopsis plants becoming shorter, flowering earlier, and generating fewer seeds (Teng et al., 2022) and *ASL18*/*LBD16*, *ASL15*/*LBD17*, *ASL20*/*LBD18*, and *ASL16*/*LBD29* have been demonstrated to be key regulators of callus induction (Fan et al., 2012). Not only are AS2/LOB family members involved in plant growth and development, but they also participate in regulating the plant’s response to abiotic stresses. For example, the ectopic expression of the apple (*Malus pumila* Mill) *MdLBD3* gene in Arabidopsis positively regulates its resistance to drought and salt stress (Liu et al., 2024), and the *PheLBD29* gene from bamboo modifies the drought stress resistance in transgenic Arabidopsis (Wu et al., 2023). *ZmLBD5* (AS2/LOB family gene) increases drought sensitivity by suppressing ROS accumulation in Arabidopsis (Xiong et al., 2022), and *VvLBD39* functions as a negative regulator of salt and drought tolerance (Chen et al., 2024).

Despite the extensive cloning and functional analysis of AS2/LOB genes in numerous species, limited information is available regarding these genes in physic nut. Physic nut (*Jatropha curcas*), a woody oil plant of the Euphorbiaceae family, holds significant application value due to its high seed oil content, tolerance to barrenness, salt, and drought, and excellent ecological and environmental protection effects (Kumar et al., 2014). In this study, a comprehensive analysis of the AS2/LOB gene family in physic nut was initially conducted based on the whole genome. Subsequently, the phylogeny, conserved domain characteristics, conserved motifs, gene structure, chromosome location, promoter cis-acting elements, and protein interaction of the identified physic nut AS2/LOB family members were analyzed. Finally, the differential expression of *JcASLs* genes in different tissues of physic nut and their expression profiles under drought and salt stress conditions were further investigated. This study aims to provide a theoretical foundation and reference information for the gene cloning and functional research of the AS2/LOB family members in physic nut, thereby offering gene resources for the breeding of salt-tolerant and drought-tolerant physic nut varieties.

## Materials and methods

### Identification of AS2/LOB gene family members in physic nut

To screen the candidate gene sequences of AS2/LOB in physic nut, the identified AS2/LOB protein sequences of Arabidopsis were downloaded from the NCBI database. Subsequently, the Arabidopsis AS2/LOB protein sequences were subjected to homologous comparison with the protein sequences of the physic nut genome using TBtools software, with the E-values set at < 0.01. A total of 28 candidate AS2/LOB proteins of physic nut were identified. Furthermore, the hidden Markov model (HMM) file of the AS2/LOB protein domain (PF00010) was downloaded from the Pfam database (http://pfam.xfam.org/). The HMMER v3.0 software was utilized to search for the AS2/LOB protein sequence in physic nut. The conserved domains of the 28 candidate physic nut AS2/LOB protein (JcAS2/LOB) sequences were confirmed using the SMART tool (http://smart.embl-heidelberg.de). All of them encompassed the AS2/LOB domain.

### Analysis of the physicochemical properties of AS2/LOB family genes in physic nut

The identified physic nut JcAS2/LOB protein sequence was subjected to a tBlastN similarity search against the physic nut genome database (accessible from DDBJ/EMBL/GenBank under the accession number AFEW00000000). Subsequently, the corresponding gene sequence, open reading frame sequence, mRNA sequence, and coding sequence were downloaded from the NCBI database. The online tool ProtParam (http://web.expasy.org/protparam/) provided by ExPaSy was utilized to analyze the basic parameters of the physic nut AS2/LOB protein sequence, such as the number of amino acids, theoretical molecular weight (Mw), isoelectric point (pI), and amino acid composition.

### Analysis of gene structure and conserved motifs of AS2/LOB family members

The CDS sequence of JcAS2/LOB genes was compared with the corresponding genomic sequences of these genes by employing the Spidey tool of GenBank (https://www.ncbi.nlm.nih.gov/) to determine the structure of introns and exons of these genes, and the gene structure diagram was drawn using GSDS (Gene Structure Display Server, https://gsds.gao-lab.org/) (Hu et al., 2015). The conserved motifs of the AS2/LOB gene family in physic nut were analyzed using the MEME online website (Multiple EM for Motif Elicitation) (https://meme-suite.org/meme/) with the following parameters: the site distribution was set as zero or one occurrence (of a contributing motif site) per sequence, the number of predicted motifs was set at 14, and the motif width was set within the range of 6 to 60 amino acids. The results of the conserved motifs analysis were visualized by means of Tbtools (Chen et al., 2023).

### Phylogenetic and chromosomal localization analysis of AS2/LOB proteins

The amino acid sequences of the AS2/LOB gene family in physic nut were compared and analyzed using DNAMAN software, with all parameters set to default values. The amino acid sequences of the AS2/LOB gene family in physic nut and Arabidopsis were compared by means of ClustalW in MEGA 10 software (Kumar et al., 2008), and the phylogenetic tree was constructed via the Neighbor-Joining (NJ) method, with the bootstrap value set at 1000 and all other parameters set to default values. The chromosome location was determined based on the genetic linkage map of physic nut constructed by Wu et al., and the gene location map was drawn using MapChart (Version 2.1).

### Analysis of cis-acting elements in the promoter region of JcAS2/LOB genes

The promoter sequences of 2000 bp upstream of the start codon ATG of each JcAS2/LOB gene were acquired through BlastN similarity search of the physic nut genome based on the CDS sequence of AS2/LOB. The cis-acting elements of the JcAS2/LOB genes (JcASLs) promoter were predicted by means of the PlantCARE online website (http://bioinformatics.psb.ugent.be/webtools/plantcare/html/). The results of the promoter cis-acting element analysis were visualized using TBtools.

### Analysis of AS2/LOB protein interaction in physic nut

The protein interaction analysis of the 28 identified physic nut AS2/LOB proteins was conducted using the STRING website (https://cn.string-db.org/). Arabidopsis was selected as the model plant. It was verified that the 28 Jatropha curcas AS2/LOB protein sequences corresponded to the proteins in Arabidopsis thaliana, and the protein interaction results were inputted.

### Preparation of plant material

The sterilized physic nut seeds were planted in blue pots with a sand:vermiculite mass ratio of 3:1 and placed in a temperature-controlled greenhouse for growth under the following conditions: a temperature of 30°C, a relative humidity of 60%, and a 16 h light/8 h dark cycle. When the physic nut seedlings reached the 6-leaf stage, the roots, stems, leaves, and seeds 16 d and 35 d after pollination were selected for tissue-specific expression analysis. For the salt stress treatment, the 6-leaf-stage physic nut seedlings were directly watered with Hoagland solution containing 150 mM NaCl, while the control group was watered with Hoagland solution. However, for the drought stress treatment, irrigation was ceased for the 6-leaf-stage physic nut seedlings. Samples were collected at 0 h, 2 h, 2 d, and 4 d for the salt treatment and 2 d, 4 d, and 7 d for the drought stress, respectively, with three biological replicates. The leaves were harvested from the fourth leaf from the top to the bottom, immediately frozen in liquid nitrogen, and stored in a -80°C ultra-low temperature refrigerator.

### RNA extraction and qRT-PCR analysis

The RNA from various tissues of physic nut was extracted using the Plant RNA Dual Column Kit (Polysaccharide Polyphenol) (Magen, http://magentec.com.cn/), and the extracted RNA was reverse transcribed into cDNA using the FastKing cDNA First Strand Synthesis Kit. Fluorescence quantitative PCR analysis was conducted using the LightCycler 480 quantitative PCR system and a 20 μL real-time PCR reaction system (10 μL of 2×SYBR Green Abstart PCR Mix, 2 μL of cDNA, 1 μL of each 10 μmol·L-1 forward and reverse primers, and 6 μL of ddH_2_O). The quantitative PCR program was as follows: 95°C for 10 min; 95°C for 15 s, 53°C for 30 s, for 40 cycles, and fluorescence signals were collected during annealing. The relative expression of genes was calculated by employing the 2^-ΔΔCt^ method. All primers used in this study were listed in **Supplementary Table S1**. All gene-specific primers were designed by using the Primer Premier 5.0 software package (http://www.premierbiosoft.com/primerdesign/) in accordance with the following criteria: PCR amplicon lengths ranging from 100 to 200 bp, Tm of 60 ± 1°C, and GC contents ranging from 45% to 60%, based on the sequences of the C-terminal regions of *JcASL* genes.

### Statistical analysis

Duncan tests were used to assess the significance of differences in measured variables between the materials with the SAS software package version 9.

## Results

### Identification of AS2/LOB gene family members in physic nut

To identify the members of the AS2/LOB family in physic nut, 42 protein sequences of AS2/LOB from Arabidopsis were employed as query sequences for BLAST alignment against the genome of physic nut. Additionally, the HMM AS2/LOB gene model was also utilized to detect AS2/LOB family members that might have remained undetected by BLAST alignment. After eliminating sequences lacking the AS2/LOB conserved domain, a total of 28 AS2/LOB proteins were identified in physic nut. According to the location order of the 28 AS2/LOB proteins on the physic nut chromosome, these proteins were named JcASL01 to JcASL28. The physicochemical properties of all identified AS2/LOB (JcASL) proteins, encompassing the number of amino acid sequence, the length of the open reading frame length (ORF), the isoelectric point (pI), and the molecular weight (MW), were analyzed and presented in **Supplementary Table S2**. The lengths of the open reading frames of the 28 *JcASL* genes ranged from 133 bp (*JcASL8*) to 349 bp (*JcASL4*), corresponding to the range of encoded amino acids from 402 to 1050 amino acid. In addition, the molecular weights of these proteins ranged from 15.02 kDa (JcASL8) to 39.22 kDa (JcASL4), and the theoretical isoelectric points ranged from 5.1 (JcASL23) to 9.13 (JcASL18). The isoelectric points of 17 JcASL proteins were less than 7.0, indicating that the majority of JcASL proteins were acidic proteins.

### Analysis of the amino acid sequence of the conserved AS2/LOB domain

**Figure 1:**
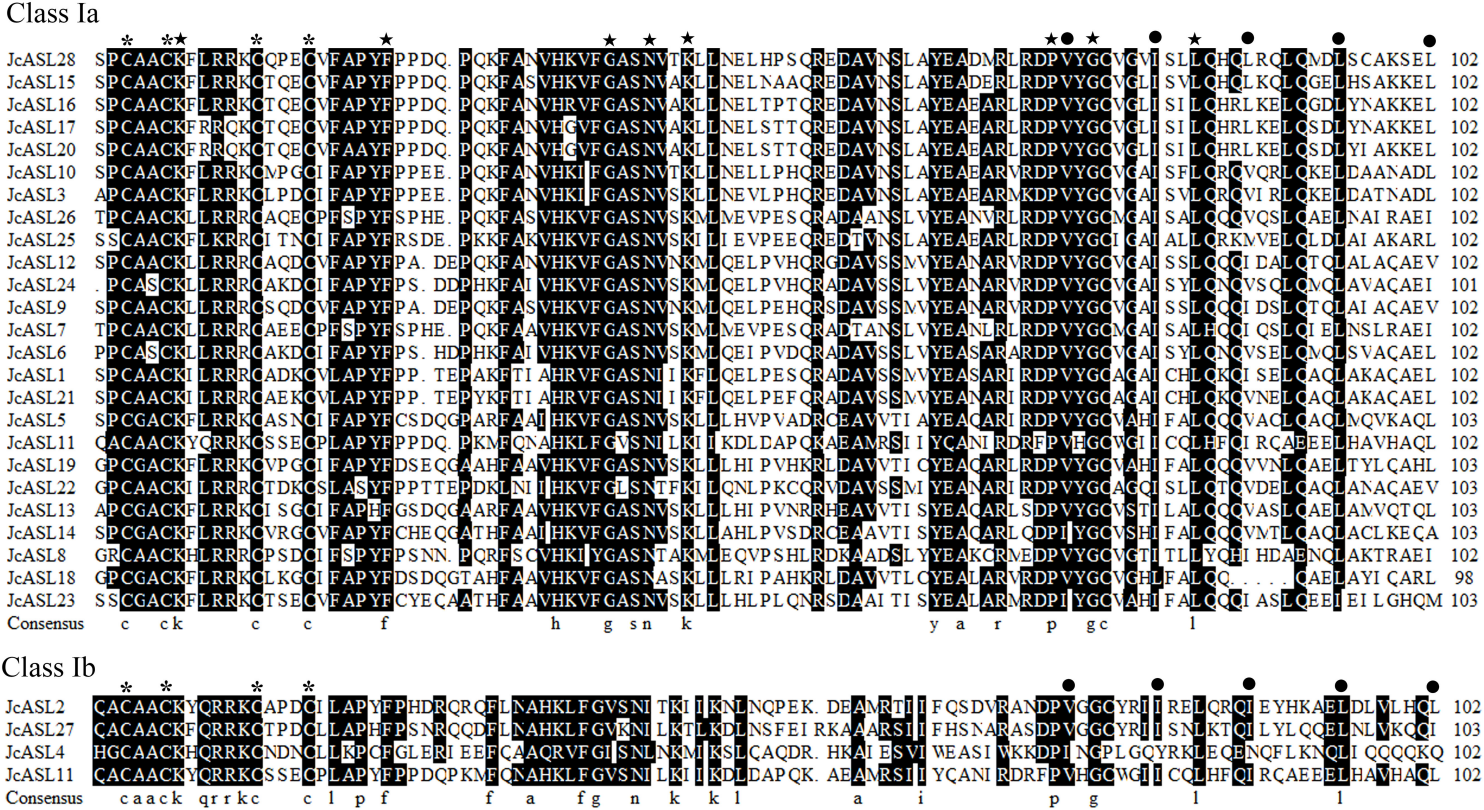
Amino acid sequence analysis of the conserved domain of AS2/LOB in the AS/LOB family of physic nut. The consensus sequence of the C motif and the hydrophobic residues located in the leucine zipper-like sequence are indicated by asterisks and black dots, respectively. The five-pointed star indicates that the amino acid residue is conserved in all AS2/LOB proteins.

Studies have shown that AS2/LOB proteins are characterized by the presence of AS2/LOB conserved domains at the N-terminus. Hence, the conserved characteristics of the AS2/LOB domain of the deduced JcASL proteins were further analyzed using DNAMAN software, as depicted in **Figure 1**. The results of our comparative analysis indicated that the CX2CX6CX3C sequence (where X represents an unconserved residue, and Arabic numerals represent the quantity of non-conserved residues), named the C-motif, was completely conserved in the conserved domains of all identified physic nut AS2/LOB proteins. Besides the C-motif, the leucine-zipper-like sequence was also highly conserved among the 28 identified JcASL proteins. More than half of the physic nut AS2/LOB proteins contained the following conserved sequences: PCAACKXLRRKCX3C within the C-motif; FAPYFX5PX2FAXVHKVF between the C-motif and the leucine-zipper-like; YGCXG and LQXQ within the leucine-zipper-like (where X represents an unconserved residue, and Arabic numerals represent the quantity of non-conserved residues). Additionally, amino acid residues K7, F23, G39, N43, K45, P73, G76, L84 (where Arabic numerals represent the positions of amino acid residues) were completely conserved within the conserved domains of the 28 identified physic nut AS2/LOB proteins.

### Phylogenetic analysis of AS2/LOB proteins

**Figure 2:**
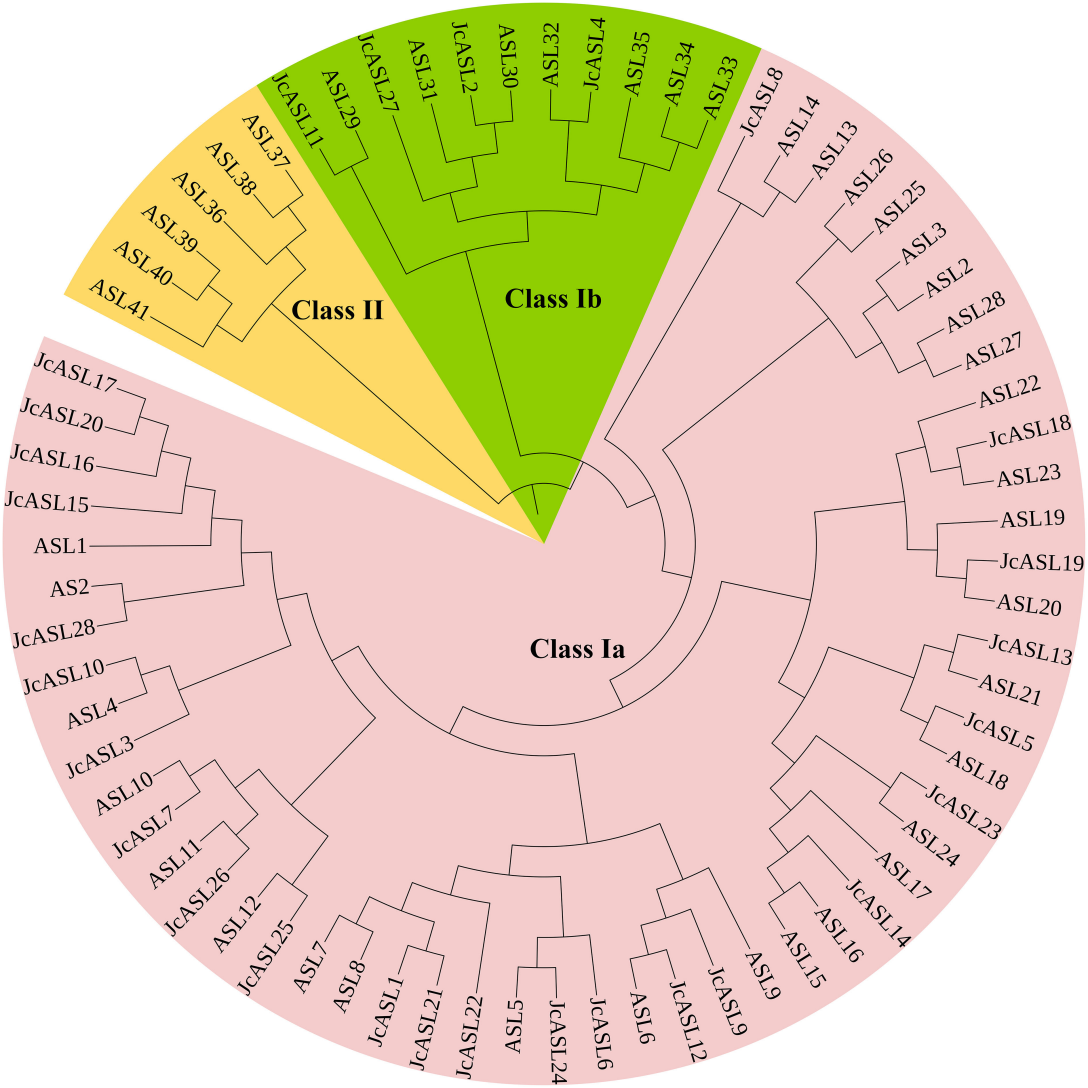
Phylogenetic analysis of AS2/LOB proteins. Neighbor-joining tree depicting the relationships among 42 AS2/LOB proteins from Arabidopsis and 28 AS2/LOB proteins from Jatropha curcas. The proteins were clustered into 2 groups, which were designated by numbers (e.g., Class I) and labeled with different alternating shades of background for enhanced identification.

To investigate the phylogenetic relationship between the AS2/LOB proteins of physic nut and Arabidopsis, a phylogenetic tree of 42 Arabidopsis AS2/LOB proteins and 28 physic nut AS2/LOB proteins was constructed using MEGA10.0 software based on the Neighbor-Joining method. Based on the previous grouping of Arabidopsis AS2/LOB proteins (Iwakawa et al., 2002), 70 AS2/LOB proteins from Arabidopsis and physic nut were classified into two groups, designated as Class I and Class II, and Class I was further subdivided into Class Ia and Class Ib (**Figure 2**). The phylogenetic results demonstrated that all 28 JcASL proteins were categorized into Class I, while no JcASL proteins were found in Class II. Four JcASL proteins (JcASL2, JcASL4, JcASL11, and JcASL27) were assigned to the Class Ia group, and 24 JcASL proteins (JcASL1, JcASL3, JcASL5, JcASL6, JcASL7, JcASL8, JcASL9, JcASL10, JcASL12, JcASL13, JcASL14, JcASL15, JcASL16, JcASL17, JcASL18, JcASL19, JcASL20, JcASL21, JcASL22, JcASL23, JcASL24, JcASL25, JcASL26, and JcASL28) were allocated to the Class Ib group. Additionally, within the phylogenetic tree, some AS2/LOB proteins from Arabidopsis and physic nut formed related sister pairs, such as ASL29 and JcASL11, ASL30 and JcASL2, ASL32 and JcASL4, ASL23 and JcASL18, ASL20 and JcASL19, ASL21 and JcASL13, ASL18 and JcASL5, ASL24 and JcASL23, ASL6 and JcASL12, ASL5 and JcASL24, ASL25 and JcASL12, ASL11 and JcASL26, ASL10 and JcASL7, ASL4 and JcASL10, and AS2 and JcASL28.

### Analysis of the gene structure of the AS2/LOB family in physic nut

**Figure 3:**
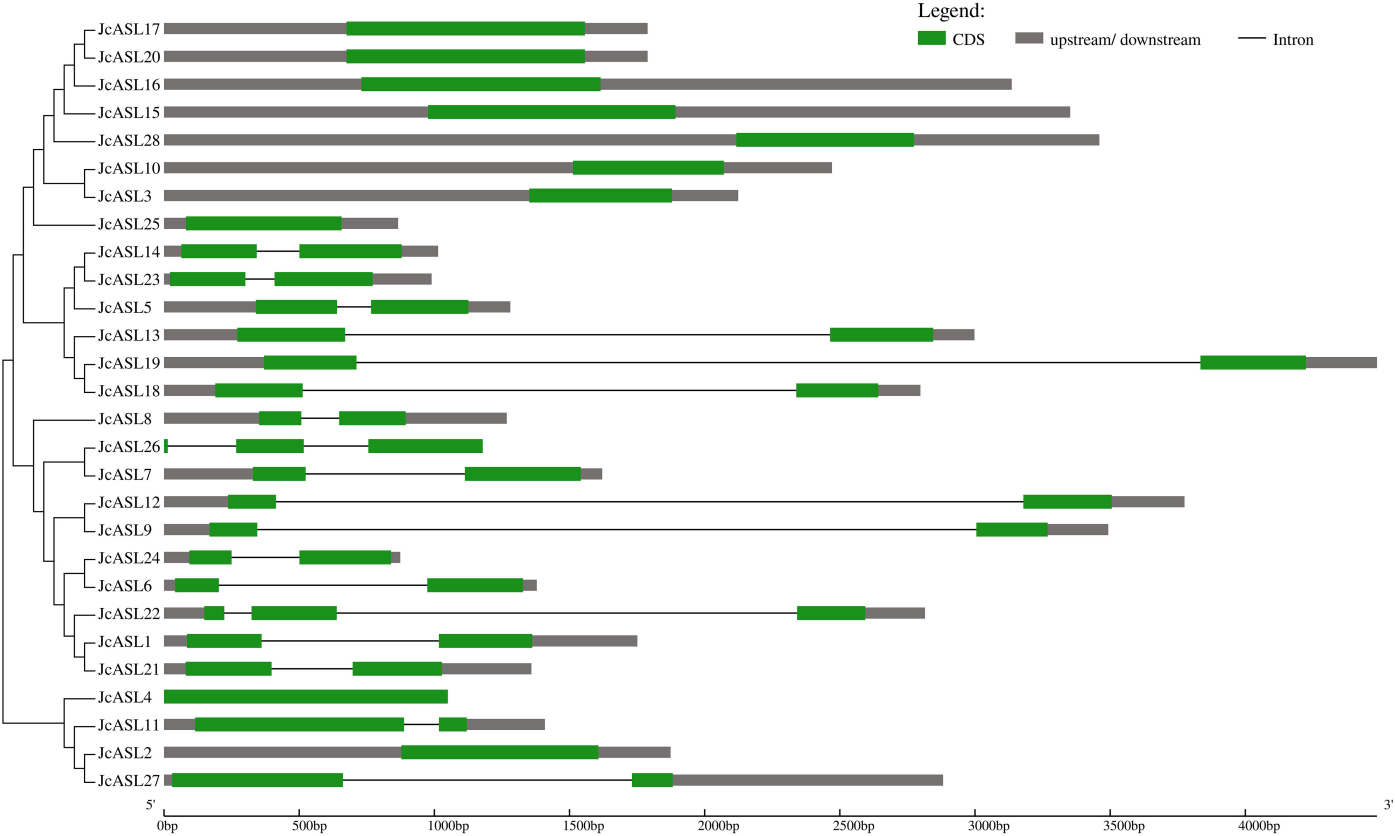
Gene structure analysis of AS2/LOB family members in physic nut. Exons and introns are presented as green boxes and thin lines, respectively. Untranslated regions (upstream/downstream) are presented as grey boxes.

In order to clarify the intron-exon structure of the *JcASL* genes, the coding region sequences of 28 *JcASL* genes and their corresponding genomic sequences were submitted to the GSDS website for gene structure analysis (**Figure 3**). In Class Ib, eight *JcASL* genes (*JcASL3*, *JcASL10*, JcASL15, *JcASL16*, *JcASL17*, *JcASL20*, *JcASL25*, *JcASL28*) did not contain introns and were clustered on one branch, while in Class Ia, only one gene (*JcASL4*) did not contain introns. Among the 28 physic nut *JcASL* genes, 18 *JcASL* genes contained 1 to 2 introns. For instance, *JcASL22* and *JcASL26* contained two introns, and the remaining 16 *JcASL* genes contained one intron.

### Analysis of conserved motifs of the AS2/LOB protein in physic nut

**Figure 4:**
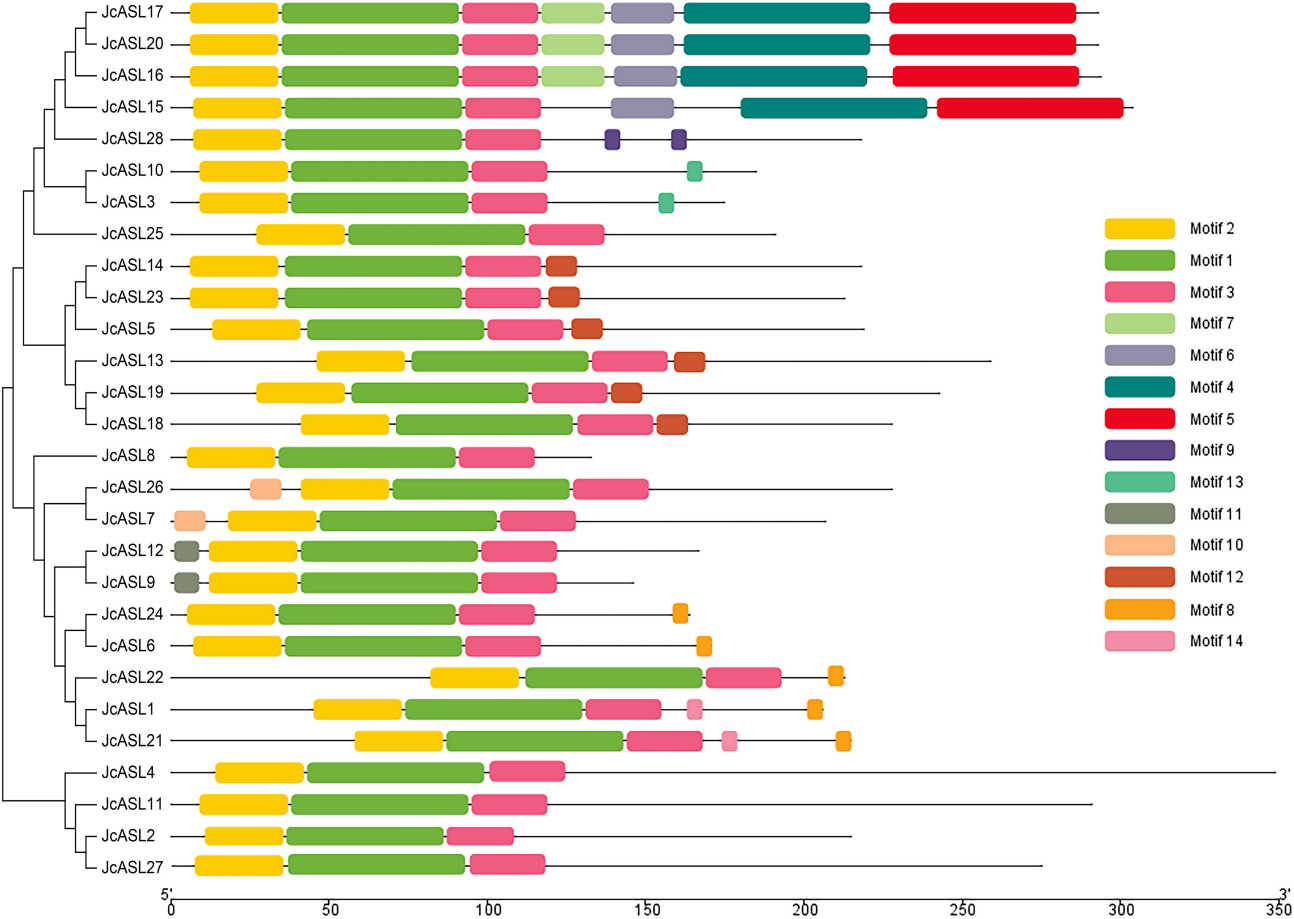
Conserved motif analysis of AS2/LOB protein in physic nut. The MEME analysis system is employed to conduct conserved motif analysis, and TBtools is utilized to visualize the results.

We further employed MEME online software to perform protein motif analysis on the amino acid sequences of 28 JcASL genes in physic nut. The results revealed that 14 conserved motifs were identified in the 28 deduced JcASL proteins (**Figure 4**). Motif 1, Motif 2, and Motif 3 were present in all 28 deduced JcASL proteins. Motif 2 corresponded to the C-motif, while Motif 3 corresponded to a leucine zipper-like sequence. Motif 4, Motif 5, and Motif 6 were only existent in JcASL15, JcASL16, JcASL17, and JcASL20, whereas Motif 7 was only found in JcASL16, JcASL17, and JcASL20. Additionally, JcASL proteins in the same branch of the phylogenetic tree exhibited similar conserved motifs. For instance, motif 10 only existed in JcASL7 and JcASL26, and motif 11 only existed in JcASL9 and JcASL12. Furthermore, Motif 8, Motif 9, Motif 10, Motif 11, Motif 12, Motif 13, and Motif 14 were only detected in Class Ib but not in Class Ia.

### Chromosome localization of *JcASL* genes in physic nut

**Figure 5:**
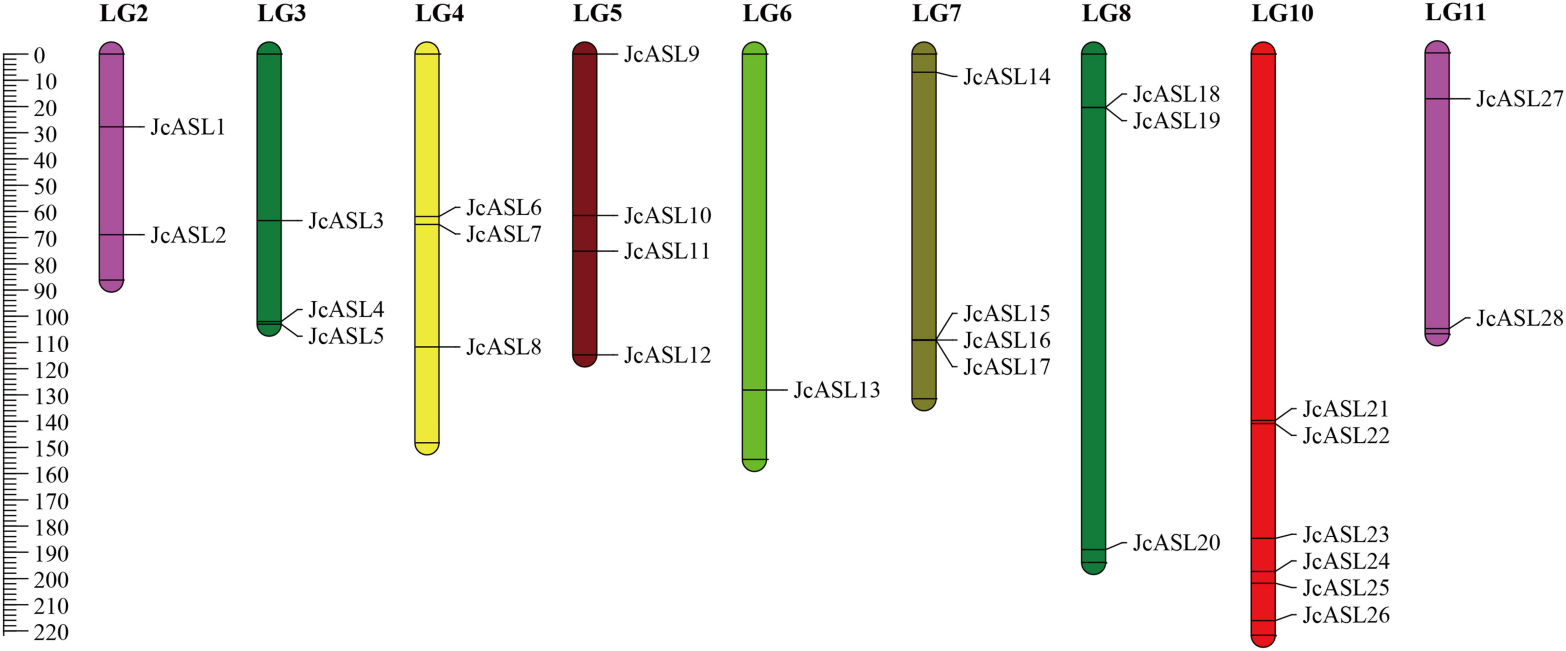
Chromosomal localization of AS2/LOB family genes in physic nut. Map chart is used to draw chromosome location maps.

Based on the high-density genetic linkage map of physic nut constructed by Wu et al., each *JcASL* gene of physic nut was located on the linkage groups (LGs) using genomic scaffold data, and the information was visualized using MapChart. The results demonstrated that the 28 *JcASL* genes were unevenly distributed across 9 LGs, and no *JcASL* genes were distributed on LGs 2 and 9 (**Figure 5**). Among the nine LGs containing *JcASL* genes, LG10 had the largest number of *JcASL* genes, amounting to 6 (*JcASL21*, *JcASL22*, *JcASL23*, *JcASL24*, *JcASL25*, *JcASL26*); LG6 had the smallest number of *JcASL* genes, being 1 (*JcASL13*). LG6 and LG11 each contained two *JcASL* genes (*JcASL1* and *JcASL2* were located in LG6, and *JcASL27* and *JcASL28* were located in LG11). LG3, LG4, and LG8 all contained three *JcASL* genes, while LG5 and LG7 both contained four *JcASL* genes. Additionally, the *JcASL* genes were mainly distributed at the lower end of LGs, accounting for 71.43%. Tandem duplication of genes is an important way for gene family expansion and the evolution of protein functional diversity. Tandem duplicates, defined as tandem repeats that are located within 50 kb from each other or are separated by less than 4 non-homologous spacer genes (Andrew et al., 2004). Two pairs of tandem duplications (*JcASL18* and *JcASL19*; *JcASL15*, *JcASL16* and *JcASL17*) of the AS2/LOB gene family were identified on LGs 7 and 8 of physic nut.

### Analysis of the promoter elements of the *JcASL* genes in physic nut

**Figure 6:**
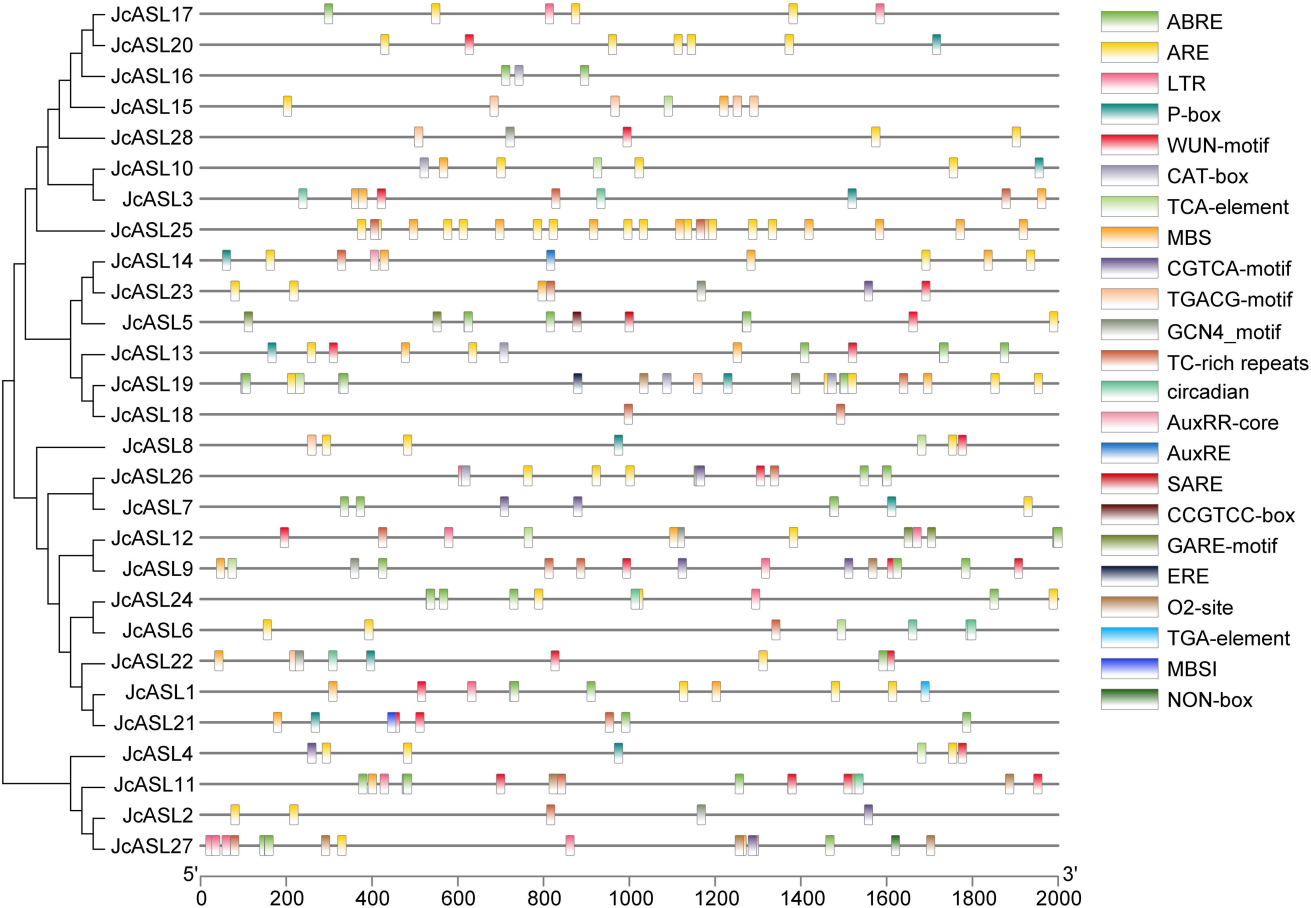
Analysis of cis-acting elements in the promoter of AS2/LOB gene in physic nut. PlantCARE is employed for promoter de-listing analysis, and TBtools is utilized for result visualization.

The 2000 bp promoter sequences prior to the start codon of the *JcASL* genes in physic nut were selected, and the cis-acting elements of the *JcASL* gene promoter were analyzed using the PlantCARE online tool and subsequently visualized with TBtools. As depicted in **Figure 6** and **Supplementary Table S3**, the results indicated that in addition to the fundamental TATA-box and CAAT-box, the *JcASL* genes also encompassed numerous cis-acting elements related to plant growth and development, phytohormone responsive, and stress. The NON-box (cis-acting regulatory element related to meristem specific activation) was exclusively found in *JcASL27*, while the TGA-element (auxin-responsive element) and the AuxRR-core (cis-acting regulatory element involved in auxin responsiveness) were respectively identified only in *JcASL1* and *JcASL14*. Stress and phytohormone responsive-related elements such as the WUN-motif (wound-responsive element), the ARE (cis-acting regulatory element essential for the anaerobic induction), TC-rich repeats (cis-acting element involved in defense and stress responsiveness), the MBS (MYB binding site involved in drought-inducibility) and the ABRE (cis-acting element involved in the abscisic acid responsiveness) were present in more than half of the *JcASL* gene promoters. *JcASL18* had the fewest elements, namely TC-rich repeats, and *JcASL19* had the maximum number of elements, amounting to 11. Furthermore, plant growth and development elements such as the CAT-box (cis-acting regulatory element related to meristem expression), the CCGTCC-box (cis-acting regulatory element related to meristem specific activation), the O2-site (cis-acting regulatory element involved in zein metabolism regulation), the GCN4-motif (cis-regulatory element involved in endosperm expression) and the circadian were also discovered in the promoter region of the *JcASL* genes. Phytohormone responsive elements such as the ABRE, the CGTCA-motif (cis-acting regulatory element involved in the MeJA-responsiveness), the TGACG-motif (cis-acting regulatory element involved in the MeJA-responsiveness), the GARE-motif (gibberellin-responsive element) and the P-box (gibberellin-responsive element) were also identified.

### Analysis of the interaction of physic nut AS2/LOB protein

**Figure 7:**
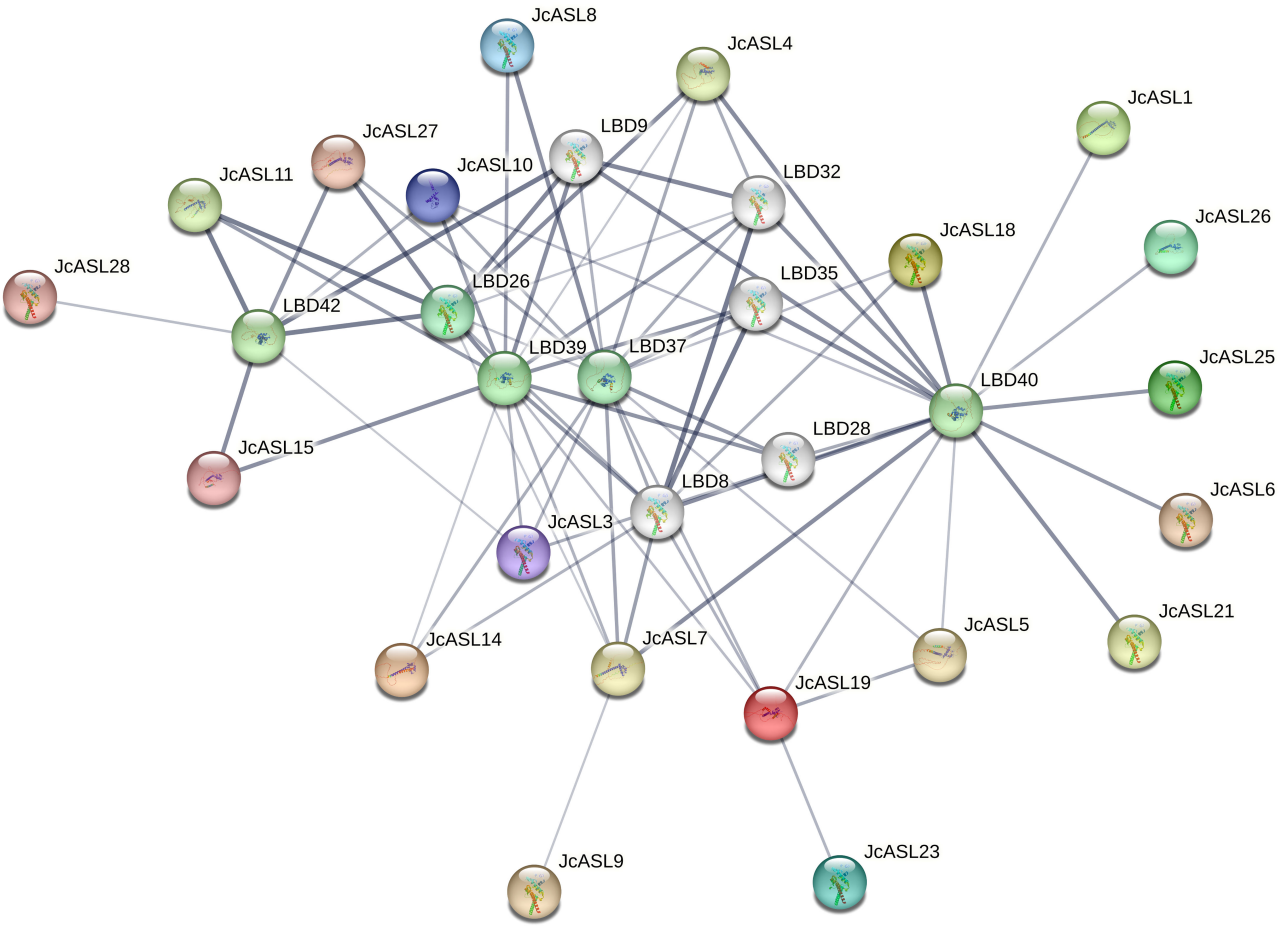
Analysis of protein interactions within the AS2/LOB family in physic nut. The thickness of the line represents the probability of interaction between proteins. The thicker the line, the higher the probability of interaction between proteins.

Employing Arabidopsis as a species model, we further predicted the AS2/LOB gene regulatory network and the interrelated genes in the protein-protein interaction network of physic nut through the STRING database, as depicted in **Figure 7**. The results indicated that there were three pairs of possible interacting proteins in the physic nut species, namely JcASL9 and JcASL7, JcASL19 and JcASL23, and JcASL19 and JcASL5. However, between physic nut and Arabidopsis, 11 physic nut JcASL proteins (JcASL1, JcASL4, JcASL5, JcASL6, JcASL7, JcASL13, JcASL18, JcASL19, JcASL21, JcASL25, JcASL26) and Arabidopsis ASL37/LBD40 proteins might interact, and 5 physic nut JcASL proteins (JcASL3, JcASL11, JcASL15, JcASL27, JcASL28) and Arabidopsis ASL36/LBD42 proteins might interact. In addition, JcASL8 may interact with both ASL39/LBD37 and ASL41/LBD39, and JcASL7 may interact with ASL28/LBD26, ASL39/LBD37, ASL41/LBD39, and ASL37/LBD40, yet the possibility of this protein interacting with ASL37/LBD40 is the highest.

### Analysis of the expression pattern of *JcASL* genes in physic nut

**Figure 8:**
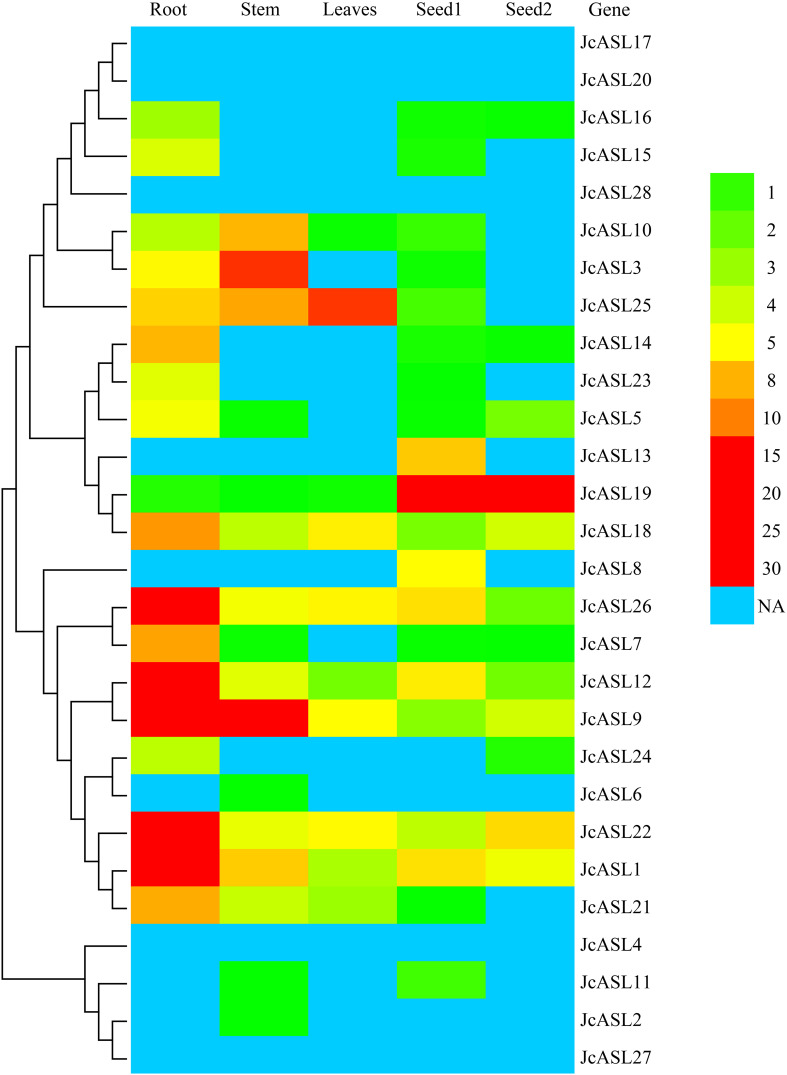
Differential expression analysis of the AS2/LOB family genes in physic nut. The color scale on the right shows the expression level. NA, Not available.

In order to clarify the potential function of the *JcASL* genes in the growth and development of physic nut, we analyzed the expression pattern of the *JcASL* genes in roots, stem cortex, leaves, and seeds at different developmental stages (seeds of 19 and 35 days after pollination) through the use of RNA-Seq data (**Figure 8**; **Supplementary Table S4**). The results revealed that five *JcASL* genes (*JcASL4*, *JcASL17*, *JcASL20*, *JcASL27*, and *JcASL28*) were not expressed in all the physic nut tissues examined, whereas 10 *JcASL* genes (*JcASL1*, *JcASL9*, *JcASL10*, *JcASL12*, *JcASL18*, *JcASL19*, *JcASL21*, *JcASL22*, *JcASL25*, and *JcASL26*) were expressed in all the tested physic nut tissues. *JcASL1*, *JcASL7*, *JcASL12*, *JcASL22* and *JcASL26* exhibited higher expression levels in physic nut roots compared to other tissues tested. *JcASL8* and *JcASL13* were expressed only in seeds among the tissues of physic nut under examination. In contrast to other tissues tested, *JcASL19* was more highly expressed in seeds, *JcASL25* was most highly expressed in leaves, and *JcASL3* and *JcASL10* were more highly expressed in stem cortex.

### Analysis of the expression patterns of *JcASL* genes in physic nut under abiotic stresses

**Figure 9:**
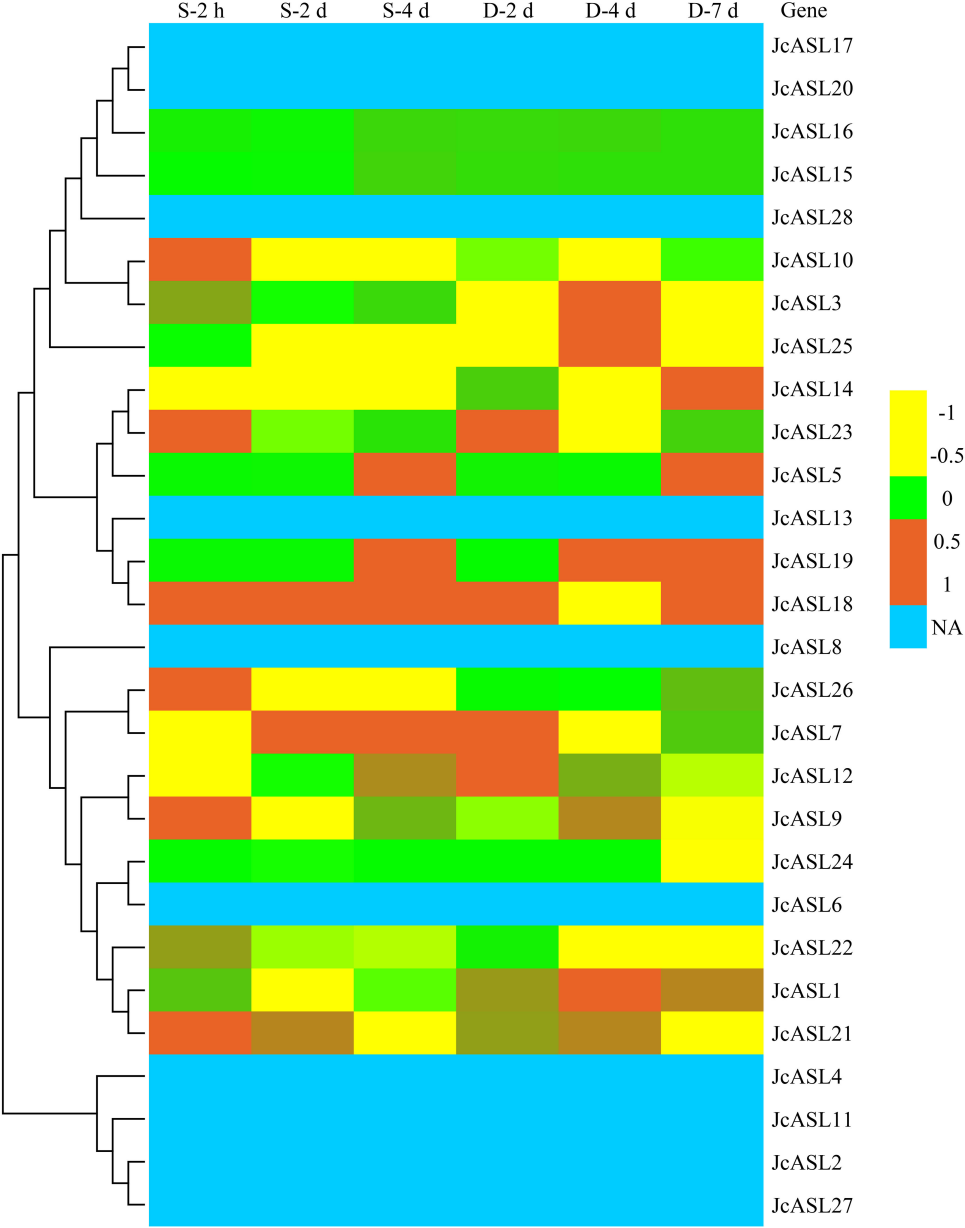
Expression of the AS2/LOB family genes in physic nut under drought and salt stress. Expression level: log_2_ ratios of signals from treated versus control roots in a heat map based on transcriptomic data, with the color scale shown on the right. NA, not available. “D” denotes drought and “S” denotes salt stress.

In order to explore the expression of *JcASL* genes in physic nut under abiotic stress conditions, we further analyzed the expression of 28 *JcASL* genes in physic nut roots under salt stress for 2 hours, 2 days, and 4 days and drought stress for 2 days, 4 days, and 7 days by utilizing RNA-Seq data (Zhang et al., 2014; Zhang et al., 2015). As illustrated in **Figure 9**, ten *JcASL* genes (*JcASL2*, *JcASL4*, *JcASL6*, *JcASL8*, *JcASL11*, *JcASL13*, *JcASL17*, *JcASL20*, *JcASL27*, and *JcASL28*) were not detectable in roots under drought and salt stress. Sixteen *JcASL* genes (*JcASL1*, *JcASL3*, *JcASL5*, *JcASL7*, *JcASL9*, *JcASL10*, *JcASL12*, *JcASL14*, *JcASL18*, *JcASL19*, *JcASL21*, *JcASL22*, *JcASL23*, *JcASL24*, *JcASL25* and *JcASL26*) responded to drought or salt stress at least under one stress condition and at least at one time point. Additionally, two *JcASL* genes (*JcASL15* and *JcASL16*) did not respond to drought and salt stress throughout all the treatment time points.

### Verification of the expression pattern of *JcASL* genes in physic nut by qRT-PCR

**Figure 10:**
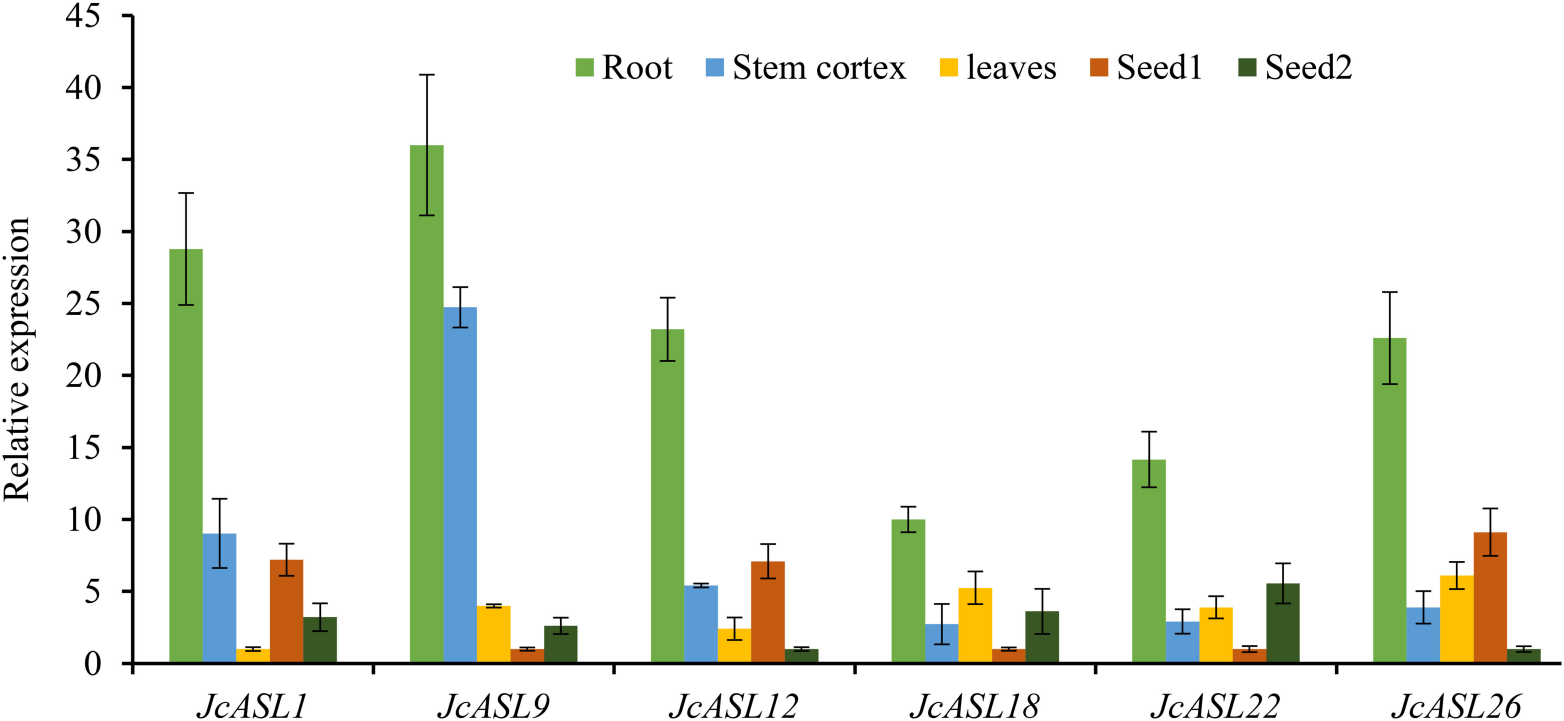
Differential expression analysis of AS2/LOB genes in different organs of physic nut by qRT-PCR. Each experiment contained three biological replicates.

**Figure 11:**
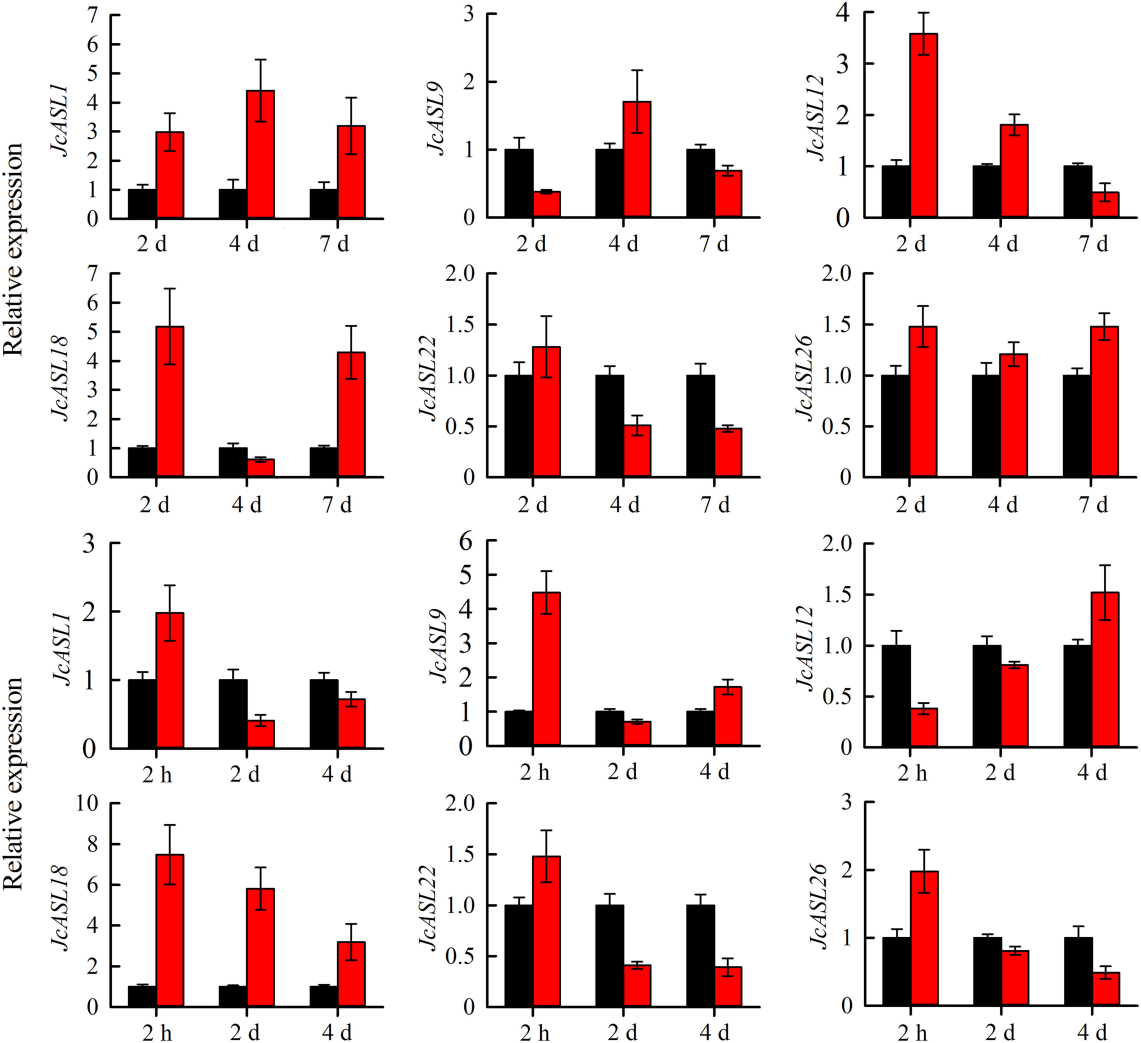
Expression analysis of AS2/LOB genes in roots of physic nut under drought and salt treatments via qRT-PCR. Each experiment contained three biological replicates. Black represents the control, and red represents salt and drought stress.

In order to verify the expression of the *JcASL* genes under normal and abiotic stress (drought and salt stress) conditions based on the RNA-Seq data, we analyzed the expression of genes in Class Ia (*JcASL1*, *JcASL9*, *JcASL12*, *JcASL18*, *JcASL22* and *JcASL26*) in different tissues under normal growth conditions and in the roots of physic nut under drought and salt stress conditions by qRT-PCR, since none of the genes in Class II were detected as expressed in the examined tissues. The results indicated that the expression of *JcASL1*, *JcASL9*, *JcASL12*, *JcASL18*, *JcASL22* and *JcASL26* in the root, stem cortex, leaf, and seed at different developmental stages in physic nut as determined by qRT-PCR was consistent with the results of RNA-seq, showing that the RNA-seq data were generally accurate (**Figure 10**). Likewise, the expression of these genes under drought and salt stress conditions detected by qRT-PCR exhibited consistent trends with the RNA-seq results (**Figure 11**). The above results further suggested that our RNA-seq data were reliable and provided valuable reference information for the further study of the function of the *JcASL* genes in physic nut.

## Discussion

Abiotic stress exerts a severe impact on the growth and development of plants. Thus, research related to abiotic stress constitutes a crucial aspect of plant adversity studies, with the AS2/LOB transcription factors being among the key regulatory factors involved in the plant’s response to adversity (Rong et al., 2024). The identification and exploration of the members of the AS2/LOB family in diverse species, particularly those that are salt-tolerant and drought-tolerant, hold significant importance. Previous studies have indicated that enhancing the expression of AS2/LOB genes can modify the ability of plants to cope with abiotic stress, and the functional verification of related genes has been confirmed in plants such as Arabidopsis (Liu et al., 2024). In the present study, 28 AS2/LOB family genes were identified from the stress-tolerant physic nut, and a subsequent analysis of the bioinformatics and expression profiles of these genes was conducted. Compared to Arabidopsis (42 AS2/LOB proteins), physic nut contains a smaller number of AS2/LOB proteins. One possible explanation could be that the AS2/LOB family genes in physic nut did not undergo a large-scale genome-wide duplication event (Wu et al., 2015), whereas such events led to the expansion of the AS2/LOB family genes in Arabidopsis (Cannon et al., 2004).

An increasing numbers of genome-wide studies on transcription factor families have revealed that the conserved motifs or functional domains within protein sequences are highly conserved within the same group or branch, such as the MYB and HD-Zip transcription factor families (Li et al., 2022; Zhang et al., 2024). These conserved motifs, which often play essential roles in regulating gene expression and determining cellular processes, can furnish valuable information for the study of the functions of unknown genes (Liu et al., 1999). Our research disclosed that the majority of the AS2/LOB proteins within the same group or branch of physic nut exhibited similar conserved motifs (**Figure 4**). In conjunction with the aforementioned previous research results, these AS2/LOB proteins with similar conserved motifs might possess similar functions in the growth and development of physic nut, which merits further investigation and exploration.

Cis-acting elements serve as vital sites for the recognition and binding of transcription factors (Li et al., 2021). The promoters of the AS2/LOB transcription factor family in physic nut contain multiple types of response elements, mainly including hormone response elements, stress response elements, and growth and development response elements (**Figure 6**). The majority of the members of the AS2/LOB gene in physic nut possess ABA and gibberellin response elements, suggesting that AS2/LOB transcription factors are extensively involved in regulating the growth and development process of physic nut. The multiple cis-regulatory elements in the upstream region of the promoter also imply the diversity of the regulatory functions of the physic nut AS2/LOB transcription factors. The identification of potential protein-protein interactions offers valuable clues regarding the functional networks in which the AS2/LOB genes are involved (Straub and Wenkel, 2017). Our findings indicate potential cross-species interactions. Specifically, 11 physic nut JcASL proteins and Arabidopsis ASL37/LBD40 proteins, as well as 5 physic nut JcASL proteins and Arabidopsis ASL36/LBD42 proteins, might interact. These potential cross-species interactions suggest the conservation of function and evolution of proteins of the same family across different plant species.

Numerous studies have demonstrated that phylogenetic trees can provide valuable reference for predicting the functions of homologous genes in different plants. For instance, genes within the same subgroup or branch are relatively conserved in aspects such as conserved motifs, expression patterns, and functions, etc (Liang et al., 2023; Tang et al., 2020). *JcERF35* from physic nut and its homologous gene *RAP2.4* in Arabidopsis belong to the A6 subgroup of the DREB family (Tang et al., 2016). The expression of both is affected by stress, and the overexpression of *JcERF35* and *RAP2.4* in Arabidopsis modifies the abiotic stress resistance of transgenic plants (Chen et al., 2023; Lin et al., 2008). Therefore, a phylogenetic tree of AS2/LOB proteins from physic nut and Arabidopsis was constructed. This result not only indicates the evolutionary relationship of AS2/LOB family proteins between Arabidopsis and physic nut but also provides a basis for predicting the functions of homologous genes in physic nut based on those of the identified Arabidopsis AS2/LOB genes. *JcASL26* and Arabidopsis *ASL11*/*LBD15* were grouped into the same branch of the phylogenetic tree (**Figure 2**). Studies have shown that *ASL11*/*LBD15* positively modulates Arabidopsis’ response to water-deficit stress and the formation of xylem vessels (Guo et al., 2020). These findings imply that *JcASL26* might be implicated in regulating the response of physic nut to drought stress and the formation of xylem vessels in physic nut. Additionally, it was notable that no JcASL proteins from physic nut were assigned to the Class II group (**Figure 2**). This finding suggests that the Class II group AS2/LOB proteins might have been retained in Arabidopsis or lost in physic nut during the evolution of the common ancestor of Arabidopsis and physic nut.

Generally, the expression profile of a gene often provides crucial information for predicting its function. By utilizing the RNA-seq data of physic nut, we analyzed the expression profile of the *JcASL* gene in the roots, stem cortex, leaves, and seeds of physic nut at different developmental stages. The highest expression of *JcASL1* and *JcASL22* was detected in roots (**Figure 8**), and the homologous genes *ASL7*/*LBD11* and *ASL8*/*LBD1* in Arabidopsis were also highly expressed in roots (Ye et al., 2021). *ASL7/LBD11 and ASL8/LBD1* have important regulatory functions in Arabidopsis root secondary growth (Ye et al., 2021). Hence, *JcASL1* and *JcASL22* might be implicated in the regulation of root growth and development of physic nut. *JcASL19* and its homologous gene *ASL19*/*LBD30* (*JLO*) in Arabidopsis are both highly expressed in seeds, and *ASL19*/*LBD30* plays a major role in Arabidopsis seed embryo development (Bureau et al., 2010). Combined with the above results, we speculate that *JcASL19* may play an important regulatory role in seed development or embryo development. *JcASL25* was highly expressed in leaves, while its homologous gene *ASL12/LBD21* has not been identified and its function remains unknown, suggesting that *JcASL25* or *ASL12/LBD21* might be involved in the regulation of plant leaf development. *JcASL10* was highly expressed in the stem cortex but scarcely detectable in roots, leaves, and seeds, implying that *JcASL10* may be involved in the regulation of stem development. In summary, the analysis of the expression profile of the AS2/LOB family genes in various tissues of physic nut can furnish valuable reference information for future studies on the functions of these genes. Previous research has demonstrated that genes belonging to the AS2/LOB family exert a significant regulatory influence on plants’ responses to abiotic stresses (Rong et al., 2024). For instance, The ectopic expression of the apple *MdLBD3* gene enhances drought resistance in transgenic Arabidopsis (Liu et al., 2024). Moreover, RNA-seq analysis under abiotic stress conditions showed that some AS2/LOB genes in *Brassica rapa* and barley were either induced or suppressed by drought or salt stress (Jiang et al., 2023; Guo et al., 2016). Similarly, in the present study, 16 *JcASL* genes resdonded to drought and salt stress (**Figure 6**). In conclusion, the results of the expression profile analysis of physic nut under abiotic stress conditions indicated that some transcription factors of the physic nut AS2/LOB family might be implicated in regulating the process of physic nut ‘ response to abiotic stresses, and their functions require further validation and exploration.

## Conclusion

In the present study, we identified 28 AS2/LOB genes in the physic nut genome and conducted a comprehensive analysis of their various characteristics and expression profiles. The differential expression of the majority of *JcASLs* genes in different tissues and the specific expression of *JcASL8* and *JcASL13* in seeds highlight the functional specificity and diversity of these genes. Moreover, the identification of 16 *JcASLs* genes responding to drought and salt stress indicates their significant role in the plant’s adaptation to abiotic stress. Overall, our research has filled a gap in the knowledge of the physic nut’s AS2/LOB genes and has laid a solid foundation for future investigations into the molecular mechanisms by which these genes regulate the plant’s response to drought and salt stress and influence its growth and development. This work also pave the way for further studies aimed at enhancing the stress tolerance and improving the overall performance of the physic nut.

## Data Availability

The datasets presented in this study can be found in online repositories. The names of the repository/repositories and accession number(s) can be found in the article/**Supplementary Material.**
